# Laboratory evolution of copper tolerant yeast strains

**DOI:** 10.1186/1475-2859-11-1

**Published:** 2012-01-03

**Authors:** Giusy Manuela Adamo, Stefania Brocca, Simone Passolunghi, Benedetto Salvato, Marina Lotti

**Affiliations:** 1 Dipartimento di Biotecnologie e Bioscienze, Università degli Studi di Milano-Bicocca, Piazza della Scienza 2, 20126 Milano, Italy; 2 Dipartimento di Biologia, Università di Padova, Via Ugo Bassi, 58/B, 35121 Padova, Italy

**Keywords:** yeast, copper, adaptation, evolutionary engineering, oxidative stress response, micronutrients

## Abstract

**Background:**

Yeast strains endowed with robustness towards copper and/or enriched in intracellular Cu might find application in biotechnology processes, among others in the production of functional foods. Moreover, they can contribute to the study of human diseases related to impairments of copper metabolism. In this study, we investigated the molecular and physiological factors that confer copper tolerance to strains of baker's yeasts.

**Results:**

We characterized the effects elicited in natural strains of *Candida humilis *and *Saccharomyces cerevisiae *by the exposure to copper in the culture broth. We observed that, whereas the growth of *Saccharomyces *cells was inhibited already at low Cu concentration, *C. humilis *was naturally robust and tolerated up to 1 g · L^-1 ^CuSO_4 _in the medium. This resistant strain accumulated over 7 mg of Cu per gram of biomass and escaped severe oxidative stress thanks to high constitutive levels of superoxide dismutase and catalase. Both yeasts were then "evolved" to obtain hyper-resistant cells able to proliferate in high copper medium. While in *S. cerevisiae *the evolution of robustness towards Cu was paralleled by the increase of antioxidative enzymes, these same activities decreased in evolved hyper-resistant *Candida *cells. We also characterized in some detail changes in the profile of copper binding proteins, that appeared to be modified by evolution but, again, in a different way in the two yeasts.

**Conclusions:**

Following evolution, both *Candida *and *Saccharomyces *cells were able to proliferate up to 2.5 g · L^-1 ^CuSO_4 _and to accumulate high amounts of intracellular copper. The comparison of yeasts differing in their robustness, allowed highlighting physiological and molecular determinants of natural and acquired copper tolerance. We observed that different mechanisms contribute to confer metal tolerance: the control of copper uptake, changes in the levels of enzymes involved in oxidative stress response and changes in the copper-binding proteome. However, copper elicits different physiological and molecular reactions in yeasts with different backgrounds.

## Background

Metal ions like copper, manganese, zinc and iron are essential micronutrients for living organisms and play a central role in the cell metabolism being the cofactors of a large number of enzymes and electron transport proteins [1]. The metabolism of copper and the mechanisms that control its intracellular concentration are the targets of intense studies since impairments in Cu level, transport and localization have been associated with several human diseases [2,3]. In fact, while copper deficiency impacts the function of key cell enzymes, Cu overload can generate highly reactive oxygen species (ROS) which produce peroxidation of membrane lipids, displacement of other metal cofactors from their natural ligands in signalling proteins [4], oxidation of proteins and cleavage of DNA and RNA molecules [5] resulting in general cellular damage. Moreover, ROS are thought to play a major role in cancer development and in aging [6]. To cope with such strict constraints, all organisms have developed complex regulatory mechanisms to maintain copper homeostasis.

Yeast cells are a good tool both for the investigation and the manipulation of copper metabolism. Studies on the accumulation of metals in edible microorganisms are of relevance for the production of functional foods enriched in micronutrients (for example the ones about the inclusion of iron, cobalt, copper and manganese in yeast cells [7]) and the industrial production of *Saccharomyces cerevisiae *biomass highly enriched with organic forms of selenium [7,8]. Yeast cells resistant to and accumulating intracellular copper have been recently patented for cleaning copper from extracellular solutions [9] and use in pro-biotic, cosmetic, dietary and nutraceuticals products [10].

It has been reported that microorganisms can acquire stress tolerance and novel metabolic abilities when exposed to the appropriate selection pressure. This approach is often reported as "evolutionary engineering", a term introduced by Butler and collaborators in 1996 [11], since it uses evolutionary principles based on the selection of random mutants arising in the microbial population either spontaneously or upon mutagenesis and has been applied for improving complex physiological properties whose genetic and physiological basis is not fully understood [12]. For example, microbial cells were recently *evolved *to improve their resistance towards multiple stresses [13], cobalt [14], iron- and sulfur-compounds [15], alcohols [16]; and to gain the ability to ferment xylose [17] and lactose [18].

*S. cerevisiae *is a powerful model organism to investigate copper metabolism and homeostasis in Eukaryotes. As a consequence, a large body of knowledge is available about Cu uptake, intracellular transport and functional role in yeast cells [19], as well as about non-enzymatic and enzymatic mechanisms of protection from ROS and oxidative stress [20].

In this study, yeast strains endowed with different natural robustness towards copper were compared with strains evolved by stepwise adaptation to tolerate high metal concentrations. We report that different and overlapping physiological and molecular responses are elicited in cells with different backgrounds to allow them to tolerate challenging conditions.

## Results

**Figure 1:**
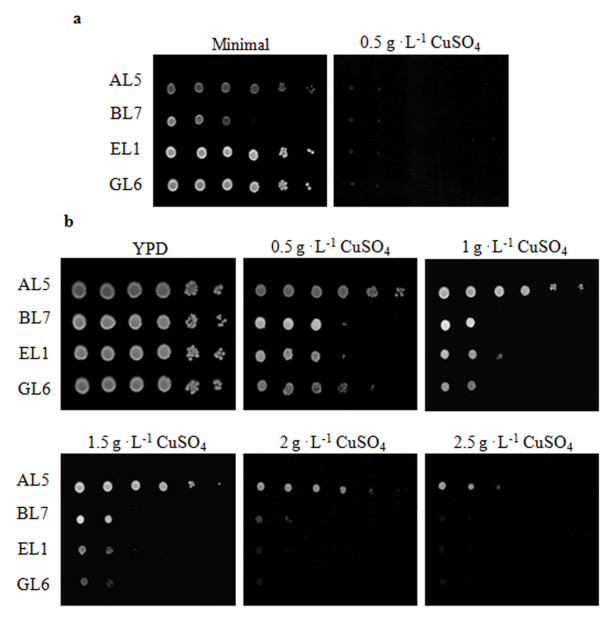
**Assay of copper tolerance in yeast strains**. Five μl of 1:10 serial dilutions were plated on minimal medium without or with 0.5 g · L^-1 ^CuSO_4 _(**a**) or YPD medium containing 0-2.5 g · L^-1 ^CuSO_4 _(**b**). Plates were incubated two days at 30°C. AL5 is the *C. humilis *strain; BL7, EL1 and GL6 are different *S. cerevisiae *strains.

Tolerance towards copper of one *Candida *and three *Saccharomyces *strains was first assessed by a drop test on minimal or rich (YPD) solid medium supplemented with increasing concentrations of copper salt (CuSO_4_) (Figure 1). In good agreement with results from other laboratories showing that the composition of the culture medium and the growth conditions affect copper sensitivity of yeast cells [21,22], we observed that on minimal medium, 0.5 g · L^-1 ^CuSO_4 _was sufficient to inhibit the growth of all yeast strains, whereas on YPD plates all of them tolerated up to 1 g · L^-1 ^CuSO_4_. However, above this concentration only *C. humilis *cells still proliferated, suggesting low copper tolerance in all strains assayed but *C. humilis *which showed a higher tolerance.

The tolerance of cells to metals is relevant both for understanding the mechanisms of defence towards stress and for the production of microorganisms enriched in a given micronutrient for biotechnological applications. We applied an evolution-based approach to improve robustness towards copper in all strains, independently from their natural background. The experimental protocol relied on the stepwise cultivation of cells in media supplemented with progressively higher concentrations of copper sulphate. At each step the culture was grown for 72 hours before withdrawing aliquots to be inoculated at a higher CuSO_4 _concentration. The starting condition was YPD + 1 g · L^-1 ^CuSO_4_, which is permissive for all strains. The following steps were in YPD + 1.5 g · L^-1 ^CuSO_4_, YPD + 2 g · L^-1 ^CuSO_4 _and YPD + 2.5 g · L^-1 ^CuSO_4_. This last one was the highest concentration applied, since above it copper salts led to acidification of the medium resulting in the precipitation of its protein components. Single colonies isolated after the last step of adaptation displayed relatively high rates of growth when directly re-inoculated in YPD + 2.5 g · L^-1 ^CuSO_4_. These cells are defined in the following as "evolved".

**Figure 2:**
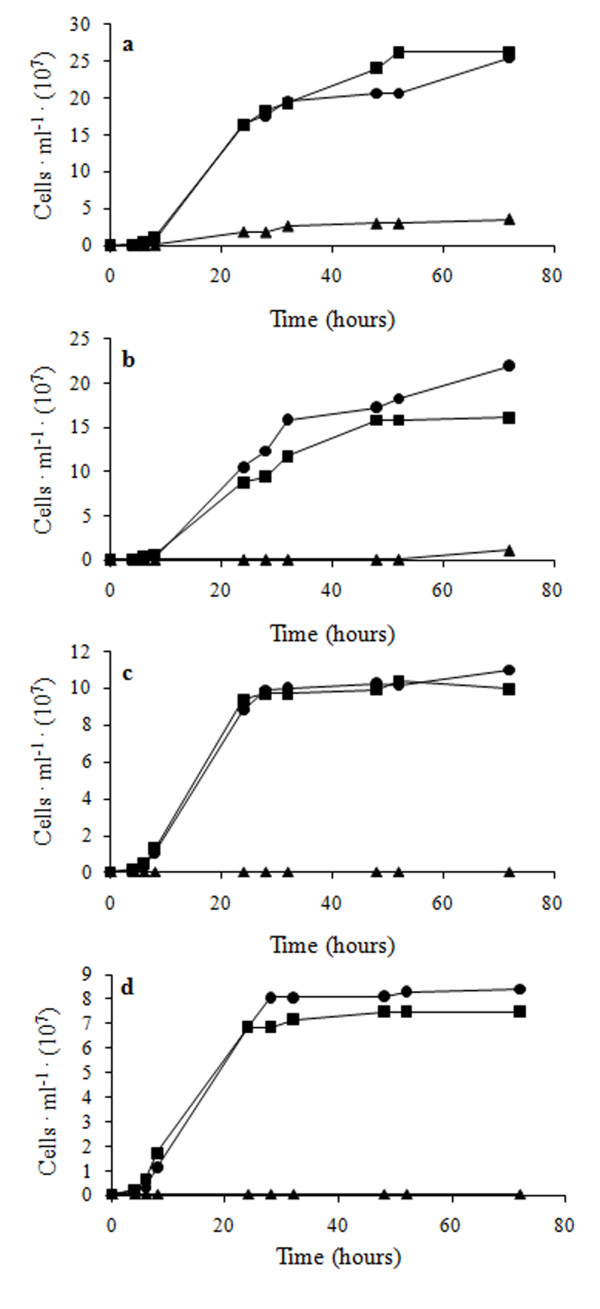
**Growth of yeast cells in YPD + 2.5 g · L^-1 ^CuSO_4_**. Evolved (black cirles), non-evolved (black triangles) and de-adapted (black squares) cells of *C. humilis *AL5 (**a**), *S. cerevisiae *BL7 (**b**), *S. cerevisiae *EL1 (**c**), *S. cerevisiae *GL6 (**d**). The values reported are averages of three replicates. Calculated standard deviations are ≤ 0.6, making error bars not appreciable.

Figure 2 compares the growth kinetics of non-evolved and evolved cells in YPD + 2.5 g ·L^-1 ^CuSO_4 _(for simplicity we will refer to this condition as "copper medium"). Among the natural strains (in the following "non-evolved"), only *C. humilis *AL5 proliferated under this condition, even though growth started after a prolonged lag phase and a very low final cell density was achieved (Figure 2 a). On the contrary, all evolved strains proliferated in copper medium and reached final biomass densities close to those observed in YPD medium, although with lower growth rate (Additional file 1). Cells subjected to 10 cycles of growth/re-inoculation in YPD without Cu (referred to as "de-adapted"), retained their ability to proliferate if re-inoculated in copper medium, showing only negligible differences when compared with the corresponding evolved strain (Figure 2 and Additional file 1). This observation suggests that copper tolerance is maintained also in absence of selective pressure. To gain more insight into the behaviour of the copper-sensitive *S. cerevisiae *strains, we compared the kinetics of growth of evolved and non-evolved cells also at 1, 1.5 and 2 g ·L^-1 ^CuSO_4 _(Additional file 2), highlighting a progressive decrease of the proliferation ability of natural cells at increasing copper concentrations. As expected, the same conditions were permissive for evolved *Saccharomyces *cells.

**Figure 3:**
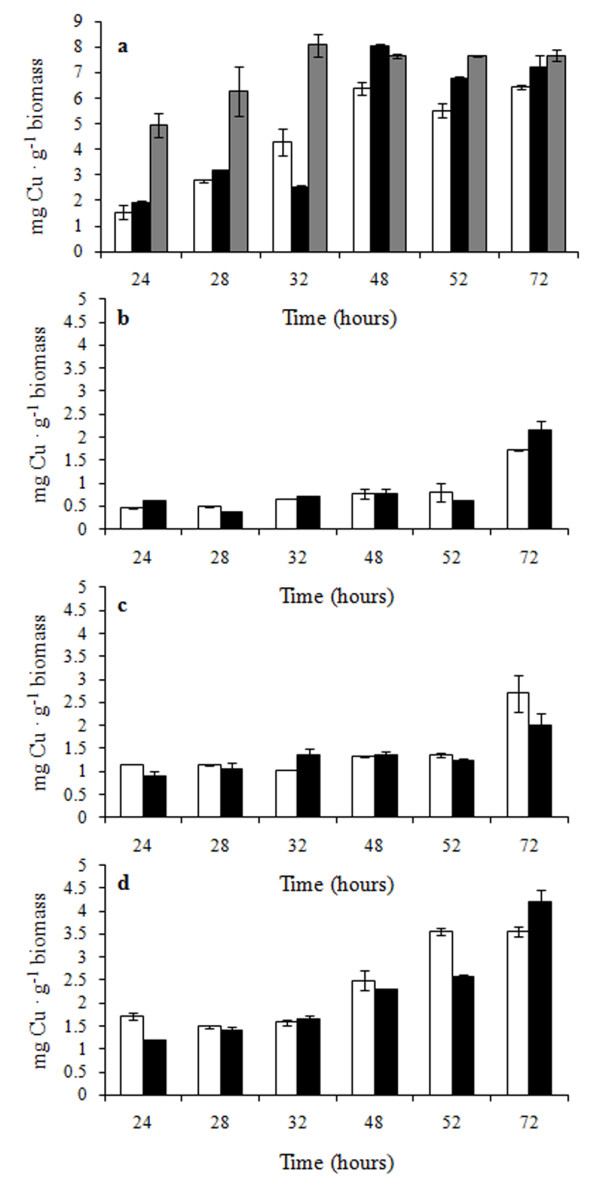
**Intracellular copper measured during growth in YPD + 2.5 g · L^-1 ^CuSO_4_**. *C. humilis *AL5 (**a**); *S. cerevisiae *BL7 (**b**); *S. cerevisiae *EL1 (**c**) and *S. cerevisiae *GL6 (**d**). White bars: evolved cells; black bars: de-adapted cells; grey bars: non-evolved cells. The amount of Cu is reported as mg · g^-1 ^biomass. Values are the average of three replicates. Note the change of scale in (**a**).

Analysis of the Cu content of *Candida *cell samples from the same cultures shown in Figure 2 revealed relatively high amounts of intracellular copper, i.e. 6.5 and 7.6 mg · g^-1 ^biomass in evolved and non-evolved cells, respectively. The kinetics of bioaccumulation was faster in non-evolved cells where Cu measured after 24 hours of growth was three times higher than in the evolved ones (Figure 3 a). In copper medium non-evolved *Candida *grew poorly and contained high copper concentration from the very beginning of the experiment, while Cu was lower in evolved cells - which proliferated at the same rate as in YPD broth. In both cases, intracellular copper kept increasing up to 48 hours and then reached a plateau. The amount of copper measured in evolved and de-adapted cells grown in copper medium was always comparable, supporting once more the hypothesis that evolutionary engineering produced stable effects.

The increase of intracellular copper in evolved *S. cerevisiae *was slower and at the end of the experiments we measured 2.1 to 4.2 mg Cu · g^-1 ^biomass (Figure 3 b, c, d). The Cu content of non-evolved and evolved *Saccharomyces *cells was compared also in a milder condition, (1 g ·L^-1 ^CuSO_4_). Whereas both kinds of BL7 cells displayed the same kinetics of copper accumulation (Additional file 3), non-evolved EL1 and GL6 showed a faster kinetic of bioaccumulation (Additional file 3) associated to a growth kinetic slower than in their evolved counterpart (Additional file 2). As in *Candida *samples, also in this case *the *behaviour of evolved and de-adapted cells was similar.

Results obtained up to this point suggested that all *Saccharomyces *strains are rather homogeneous in their response to copper, but different from the *Candida *one. Therefore, subsequent experiments aimed at highlighting possible adaptive changes were focused on a more in-depth comparison between *Candida humilis *and the only *Saccharomyces *cells strain BL7.

**Figure 4:**
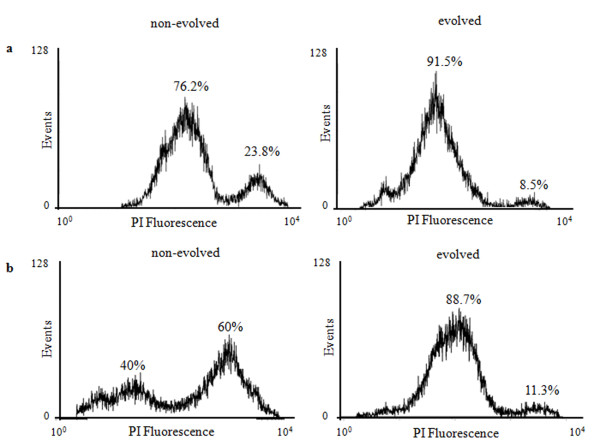
**Cytofluorimetric analysis**. Evolved and non-evolved *C. humilis *(**a**) and *S. cerevisiae *(**b**) cells. Cells were collected during exponential growth in YPD + 2.5 g ^. ^L^-1 ^CuSO_4 _and stained with propidium iodide. The x- and y-axes of the histogram display the log fluorescence intensity (PI) and the number of collected cells (events) per sample, respectively. Propidium positive cells are on the right side of the distribution whereas viable cells are on the left side. Fluorescence distributions are representative of three replicates obtained in independent experiments. This analysis was carried out with *S. cerevisiae *cells despite their extreme poor growth in copper medium thanks to the very small number of cells required.

Initially, the effect of copper on cell viability was evaluated by citofluorimetry (Figure 4), that allows to get this information at the single cell level [23]. Samples of *C. humilis *and *S. cerevisiae *cultures were harvested during exponential growth in copper and stained with propidium iodide, an intercalating agent excluded by viable cells, that can instead permeate the surface of seriously injured/dead cells [24]. In both evolved strains the percentage of propidium-positive cells was lower (8.5% for *C. humilis *and 11% for *S. cerevisiae*) than in their non-evolved counterpart (28% for *C. humilis *and 60% for *S. cerevisiae*). The percentage of propidium-positive cells grown in absence of copper (used as a control) was around 2% (*data not shown*). Altogether, these results confirm that evolution confers robustness - although not complete insensitivity - to copper. Copper was more detrimental for *S. cerevisiae *than *C. humilis *natural cells, while this difference fainted after adaptive evolution, in good agreement with the kinetics of growth reported in Figure 2.

Since copper is a strong oxidizing agent, we measured the activities of superoxide dismutase, peroxidase, glutathione peroxidase and catalase - all involved in the so-called copper-dependent oxidative stress response - in evolved and non-evolved *C. humilis *and in evolved *S. cerevisiae *cells harvested from copper medium in the exponential phase of growth. As a control, basal enzymatic activities were determined in YPD-grown cells (Table 1). In *S. cerevisiae*, exposure to copper resulted in the increase of superoxide dismutase and catalase activities, while peroxidase and glutathione peroxidase activities were only marginally affected. The picture emerging from the analysis of the copper tolerant *C. humilis *strain is different and more exhaustive, since the data set can also include the response of non-evolved cells in copper medium. In all cultivation broths, all enzymatic activities tested in natural *Candida *cells were 2 to 8 fold higher than in *S. cerevisiae*. Interestingly enough, we detected high constitutive superoxide dismutase and catalase activities in non-evolved cells, independently from the composition of the culture broth, while peroxidase and glutathione peroxidase activities were induced only by growth in copper medium. Evolution resulted in lower levels of all tested activities in both YPD and copper medium, with the most remarkable effect on superoxide dismutase. Catalase activity that was high in YPD-grown cells (both kinds of cells), strongly decreased in evolved cells grown in copper medium.

**Table 1:** Antioxidant enzyme activities in *S. cerevisiae *and *C. humilis *cells grown in YPD and in YPD + CuSO_4 _2.5 g · L^-1 ^(Cu)

		*S. cerevisiae*				*C. humilis*		
	
	non-evolved		evolved		non-evolved		evolved	
	
	YPD	Cu	YPD	Cu	YPD	Cu	YPD	Cu
SOD	20.93 ± 0	n.d	39.22 ± 0	64.14 ± 0.92	132.97 ± 17.09	117.2 ± 21.26	32.34 ± 1.08	41.1 ± 8.42
Catalase	3.2 ± 0.45	n.d	3.23 ± 0.45	11.82 ± 1.26	81.5 ± 5.17	73.76 ± 6.74	74.14 ± 11.46	27.7 ± 4.4
Peroxidase	0.001 ± 0	n.d	0.0032 ± 0	0.004 ± 0	0.011 ± 0.001	0.019 ± 0.003	0.014 ± 0	0.008 ± 0
GPO	0.041 ± 0.001	n.d	0.012 ± 0.001	0.013 ± 0.009	0.19 ± 0.024	0.534 ± 0.039	0.235 ± 0.039	0.172 ± 0.07

Activities measured were superoxide dismutase (SOD), catalase, peroxidase and glutathione peroxidase (GPO). Values, reported as U · mg^-1 ^of proteins, are average of three independent measurements. n.d.: not determined due to lack of viable cells.

**Figure 5:**
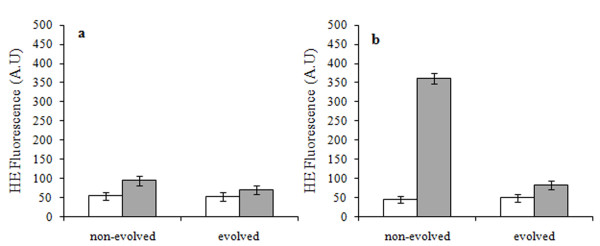
**Fluorimetric analysis of superoxide anion (OH^-•^) production**. Evolved and non-evolved *C. humilis *(**a**) and *S. cerevisiae *(**b**) cells exponentially growing in YPD (white bars) and in YPD + 2.5 g ^. ^L^-1 ^CuSO_4 _(grey bars). OH^-• ^formation is expressed as fluorescence intensity of ethidium in arbitrary units. Data presented are the mean of at least three independent analyses.

We then evaluated the production of reactive oxygen species (ROS) staining cells with dihydroethidium (Figure 5). In presence of superoxide anions in the cytosolic space, this probe is oxidized to the fluorescent product ethidium. Therefore, fluorescence intensity reports on oxidative stress. While in YPD medium the amount of ROS was low in both *Candida *and *Saccharomyces *cells, copper exposure clearly triggered oxidative stress, though with milder effects in the evolved cells. Moreover, in agreement with growth and cytofluorimetric data (Figure 2 and 4), the effect on *S. cerevisiae *was stronger than on *Candida *with ROS production three-fold higher. Evaluation of oxidative stress in *S. cerevisiae *at intermediate metal concentration (1, 1.5 and 2 g ·L^-1 ^CuSO_4_) showed that while ROS production increases with copper concentration in the non-evolved sample, it remains low in the evolved cells (Additional file 4).

**Figure 6:**
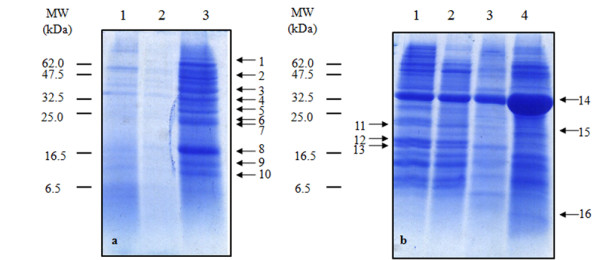
**SDS-PAGE of copper-binding proteins**. (**a**) *S. cerevisiae*. Lane 1: proteins from non-evolved cells grown in YPD; lane 2: proteins from evolved cells grown in YPD; lane 3: proteins from evolved cells grown in YPD + CuSO_4 _2.5 g · L^-1^. (**b**) *C. humilis*. Lane 1: proteins from non-evolved cells grown in YPD; lane 2: proteins from evolved cells grown in YPD; lane 3: proteins from non-evolved cells grown in YPD + CuSO_4 _2.5 g · L^-1^; lane 4: proteins from evolved cells grown in YPD + CuSO_4 _2.5 g · L^-1^.

Finally we performed a preliminary analysis of the copper-binding proteins extracted from non-evolved and evolved cells grown either in YPD or in copper medium. Samples were enriched by affinity chromatography as described in Materials and Methods and then analysed by SDS-PAGE. Equal volumes of elution fractions obtained from the same amount (800 μg) of proteins applied on the column were loaded for the electrophoretic run, therefore assuring that differences detected in the gel reflect changes in composition and content of copper-binding proteins in the starting samples. Figure 6 shows the electrophoretic analysis and Table 2 lists proteins identified by tandem mass spectrometry. The exposure of evolved *S. cerevisiae *cells to copper elicited the induction of several proteins (Figure 6a, lane 2 and 3) involved in different biochemical and metabolic functions, i.e. the pentose phosphate pathway (band 1), amino acid and sulphur metabolism (bands 2, 3 and 10), glucose metabolism (bands 4 and 7), redox reactions (bands 6 and 8), the translation machinery (bands 5 and 9) and isomerization reactions (bands 1 and 9). The same trend was detected at lower CuSO_4 _concentration (data not shown). The picture relevant to *Candida *is completely different (Figure 6 b). Non-evolved *Candida *cells react to copper repressing a number of Cu-binding proteins that would be otherwise expressed during growth in YPD (Figure 6b, lane 1 and lane 3). Among the down-regulated proteins we could identify ribosomal proteins and components of the protein translation apparatus (band 11, 13 and 16). The profile of Cu-binding proteins extracted from evolved *Candida *cells (Figure 6, lane 2 and 4) showed a massive enrichment of a protein of ~35 kDa (band 14), identified as glyceraldehyde-3-posphate dehydrogenase 3 (GAPDH). We further observed the increase of a protein of ~ 22 kDa (band 15) identified as peroxiredoxin and of a protein inducible by oxidative stress (band 12).

**Table 2:** Identification of copper-binding proteins

Band n°	Protein^a^	MW (kDa)	n° peptides^b^
1	Transketolase 1	73.87	4
	Protein disulfide-isomerase	58.53	1
2	NADP glutamate dehydrogenase 1	49.88	14
	NADP glutamate dehydrogenase 2	49.93	6
3	Aspartate-semialdehyde dehydrogenase	40.03	3
	Cystathionine- γ-lyase	42.51	4
4	Glyceraldehyde-3-phosphate dehydrogenase	35.83	4
5	Elongation factor 1-β	22.67	3
6	Uncharacterized oxidoreductase YMR226C	29.19	3
7	Triose phosphate isomerase	26.89	3
8	Cu, Zn-superoxide dismutase	15.95	3
9	Peptidyl-prolyl cis-trans isomerase	17.49	2
	40S ribosomal protein S26-A	13.72	1
10	Hypothetical protein YIL051C (or MMF1)	15.95	3
11	60S ribosomal protein L19	21.69	1
12	Hypothetical protein YDR032C	20.96	1
13	Eucaryotic translation initiation factor 5A-2	17.21	1
14	Glyceraldehyde-3-phosphate dehydrogenase 2	35.93	3
	Glyceraldehyde-3-phosphate dehydrogenase 3	35.83	4
15	Peroxiredoxin TSA1	21.69	1
16	Ubiquitin-40S ribosomal protein S31	17.43	2

^a ^ESI-MS indicates that bands 1, 2, 3, 9 and 14 contain more than one protein

^b ^number of peptides analyzed by ESI-MS

## Discussion

In this work, we obtained copper-enriched and copper-tolerant yeasts through a number of generations smaller than reported in similar recent works [13,14,25]. This might be explained on the basis of a different experimental set-up in which relatively few rounds of selection were applied, but with longer cultivation times (72 h) and wider intervals of metal concentration (increases of 0.5 g. L^-1 ^at each round). The effectiveness of the method of evolution is substantiated by the preservation of metal tolerance in absence of selective pressure (de-adapted cells), meaning that stable molecular changes occurred. This view is corroborated by the amplification of the CUP1 gene, which encodes for a metallothionein, detected in evolved *S. cerevisiae *cells (Adamo et al., in preparation). However, it is possible that other adaptive (transient) modifications contribute to the increased resistance.

In the frame of this complex picture, the comparison of yeasts differing in their robustness towards copper allowed us to investigate physiological differences involved in natural and acquired copper tolerance and to obtain some preliminary information about the molecular determinants of this trait. Taken together, our results hint at the concurrence of different mechanisms that we briefly summarize and discuss in the following.

*i) Copper uptake*. *C. humilis *cells can grow in copper medium due to their natural tolerance that can be further improved *via *evolutionary engineering. This feature allowed to compare the bioaccumulation of intracellular Cu in non-evolved and evolved cells at the highest copper concentration used (2.5 g ·L^-1 ^CuSO_4_), showing that copper is lower in these latter. On this basis we hypothesize that one of the mechanisms of robustness might rely on hindering metal uptake. Consistently, the toxicity of Cu incorporated in the non-evolved strain (mainly in the first hours of growth) might account for the growth impairment and the increase in the rate of propidium positive cells we observed. In this light, the variability in copper sensitivity between *Candida *and *Saccharomyces *cells might depend (at least partly) on a different ability to limit copper uptake and its overload. Such a mechanism has been reported to protect *S. cerevisiae *cells from copper [26] cadmium [27] and cobalt [14] and points to a central role of the plasma membrane [28-30] and of the cell wall [31] in the onset of tolerance to heavy metals.

*ii) Antioxidative enzymes and ROS production*. Our results indicate that the biochemical bases of copper resistance can be deeply different among yeast species. In *S. cerevisiae*, evolution of copper tolerance is associated with the increase of antioxidative activities, as already documented by others [32], and with a reduced production of ROS. On the contrary, non-evolved cells suffer severe oxidative stress, as showed by the complete inhibition of their growth and the huge percentage of propidium positive cells. Basal activities of most detoxifying enzymes are higher in *C. humilis *than in *S. cerevisiae *cells, fact that could partly explain the natural copper tolerance of *Candida *cells and the reduced ROS production. The response of evolved *Candida *cells to copper is intriguing since our results show a reduction in the production of ROS, a generalised decrease of the antioxidative activities, and non-responsiveness to copper (compare values reported for evolved cells in YPD and copper medium). Indeed, in evolved cells superoxide dismutase is always lower than in the original strain, independently from the presence of copper. We would assume that the different activity profiles and their re-shaping upon evolution might reflect different and peculiar defence mechanisms responsible for both natural and acquired copper tolerance in *Saccharomyces *and *Candida *strains.

*iii) Cu-binding proteins*. The hypothesis above is further corroborated by the observation that the amounts of soluble Cu-binding proteins extracted from non-evolved and evolved cells are different too. Over-expression of Cu-binding proteins is consistent with their role in the primary response to copper exposure and results in copper tolerance [33]. We are aware that the enrichment procedure used might lead to overestimation of some proteins that carry modifications such as thiolation [34,35] or sequence motives that increase their affinity for the chromatographic resin or to underestimation of low-affinity proteins. Nevertheless, the differences detected between the two yeasts are marked and relevant proteins identified are in agreement with literature data reported by others. Among Cu-binding proteins, enzymes involved in sulphur metabolism (i.e. cystathionine-γ-lyase and aspartate-semialdehyde dehydrogenase) were over-expressed by evolved *S. cerevisiae *cells during growth in copper medium, suggesting that copper might redirect the metabolic flux towards the production of GSH to balance the redox equilibrium. The over-expression of glycolytic enzymes such as triose phosphate isomerase and GAPDH3 might be a consequence of the reconfiguration of the glycolytic flux, a mechanism reported to regulate the response to oxidative stress in human [36], plant [37] and yeast [38] cells. Furthermore, the increase of GAPDH3 is consistent with the role of this protein as a sensor of oxidative stress in DNA repair [39] and apoptosis [40,41]. GAPDH3 increase is also the most remarkable effect triggered by copper in evolved *C. humilis*. Besides, these cells are enriched in the peroxiredoxin Tsa1 [42,43], known to act as an antioxidant against ROS [44] and to protect actively translating ribosomes from stress conditions [45]. On this basis we propose that in evolved *Candida *cells the inhibition of protein synthesis associated with oxidative stress [46] occurs in an attenuated form in comparison to non-evolved cells, that show a strong decrease of Cu-binding proteins, without any remarkable down regulation of the ribosomal proteins and other components of the protein translation apparatus.

## Conclusion

The comparison between yeast cells naturally resistant or experimentally evolved to tolerate high copper concentration reported in this work supports the view that copper tolerance is due to multiple responses relying on different physiological and macromolecular changes. Yeasts endowed with copper tolerance and able to accumulate metal ions can find application in the biotechnology field for example for bioremediation or as dietary supplement, being these "GRAS organisms" valuable sources of microelements in organic form. Moreover, the comprehension of physiological and molecular responses of microorganisms to metal stress and of the mechanisms triggered during evolution of tolerance could help in the identification of biomarkers for ecotoxicological studies.

## Methods

### Yeast strains and growth conditions

Yeasts used in this study were isolated from sourdough. Strains were identified by Random Amplification of Polymorphic DNA-PCR (RAPD-PCR) and designated as *Candida humilis *AL5, and *Saccharomyces cerevisiae *BL7, EL1 and GL6 (Veneto Agricoltura - Istituto per la Qualità e le Tecnologie Agroalimentari - VI). Growth was on YPD medium [2% (w/v) glucose, 1% (w/v) yeast extract, 2% (w/v) tryptone] or on minimal medium [2% (w/v) glucose, 0.67% (v/v) yeast nitrogen base]. Solid media contained 2% (w/v) agar.

Copper tolerance was tested on cells grown shaking over night in 3 mL of liquid YPD at 30°C and subjected to serial dilutions in physiological solution [0.9% (w/v) NaCl]. 5 μL aliquots were spotted on either YPD or minimal medium plates containing CuSO_4 _and incubated at 30°C for 2 days.

Adaptative evolution was performed stepwise starting from cells taken from a fresh culture on agarized YPD and grown overnight at 30°C in 3 mL of liquid YPD + 1 g · L^-1 ^CuSO_4_. In the subsequent steps, 5 × 10^5 ^cells from stationary cultures were inoculated and cultivated for 72 hours in fresh medium containing each time increasing concentrations of copper (1.5 - 2 - 2.5 g · L^-1^). Single colonies were isolated from the culture on YPD + 2.5 g · L^-1 ^CuSO_4 _by plating on solid YPD and re-inoculated in liquid YPD + 2.5 g · L^-1 ^CuSO_4_. To assess the endurance of metal tolerance, evolved cells were subjected to 10 cycles of inoculation-growth in fresh YPD medium without copper (de-adaptation) prior to be cultivated again in YPD + 2.5 g · L^-1 ^CuSO_4_.

Growth of non-evolved and evolved *S. cerevisiae *cells (BL7, EL1 and GL6) was assayed in liquid YPD medium supplemented with intermediate copper concentrations (1, 1.5, 2 g, · L^-1 ^CuSO_4_).

Growth was determined as the increase of cells number using a cells counter (Particle Count & Size Analyzer, Beckman Coulter).

### Copper determination

Cells were harvested from 2 mL culture by centrifugation at 10,000 *g *for 10 min, washed twice with de-ionized water and at least four times with 10 mM citric acid in 0.5% (w/v) NaCl to remove copper ions adsorbed on the cell surface. The biomass was dried by Max-Dry Iyo (Heto) for 30 min, re-suspended in 300 μL of 20% (w/v) trichloroacetic acid (TCA), transferred to a 2 mL screw cap tube containing 100 μL of glass microbeads and subjected to mechanical lysis by three cycles of 20 sec at maximum speed with a Fast Prep^® ^- FP120 (Bio101-Savant). The crude extract was clarified by centrifugation at 10,000 *g *for 10 min and the supernatant transferred to a new tube. Copper was quantified according to Brenner and Harris [47] adapted as follow. The clarified crude extract was diluted in 500 μL of de-ionized water and added to 100 μL of 1% (w/v) ascorbic acid and 400 μL of "BCA reagent" [0.006% (w/v) 2,2'-biquinoline-4,4'-dicarboxylic acid disodium salt hydrate (Fluka), 3.6% (w/v) NaOH, 15.6% (w/v) Hepes]. After 2 min incubation at room temperature, absorbance was recorded at 354 nm with a spectrophotometer Ultraspec 1000 (Pharmacia Biotec). The amount of copper is referred as mg Cu · g^-1 ^dry biomass.

### Flow cytometry analysis

Samples containing 10^7 ^cells were withdrawn during the exponential growth phase. Cells were harvested by centrifugation, washed twice with deionized water and four times with 10 mM citric acid in 0.5% (w/v) NaCl. Cells were washed with 1 mL of PBS (3.3 mM NaH_2_PO_4_, 6.7 mM Na_2_HPO_4_, 127 mM NaCl, 0.2 mM EDTA) and stained with 1 mL of the fluorescent probe propidium iodide (5 ng ^. ^mL^-1^). Stained cells were sonicated for 15 sec and then analyzed using a Cell Lab Quanta™SC flow cytometer (Beckman Coulter) equipped with a diode laser (excitation wavelength 488 nm, laser power 22 mW). The fluorescence emission was measured through a 670 nm long pass filter (FL3 parameter) in logarithmic mode for propidium iodide (PI) signal. Not stained and ethanol-treated samples were used as controls. The sample flow rate during analysis did not exceed 500 cells sec^-1^. A total of 2 × 10^4 ^cells were measured for each sample. Data analysis was performed with WinMDI 2.8 software, build#13 01-19-2000 (Purdue University, Cytometry Laboratories http://facs.scripps.edu/software.html).

### Preparation of cell-free extracts

Cells from exponential cultures were harvested by centrifugation at 4,900 *g *for 10 min and washed twice with cold deionised water and four times with cold 10 mM citric acid in 0.5% (w/v) NaCl. The cell pellet was finally re-suspended in 0.5 M Tris-Cl pH 8.5, 0.25 M EDTA pH 8.4 added with protease inhibitor cocktail (Sigma) and mechanically disrupted using glass microbeads. Cell debris was removed by centrifugation at 700 *g *for 10 min and the clarified crude extract was used for enzymatic analyses. The protein concentration in cell-free extracts was estimated according to Bradford [48] using bovine serum albumin as the reference.

### Enzyme assays

Enzyme activities were measured on cell-free extracts by spectrophotometric assays. Activities were expressed as Units · mg^-1 ^proteins. Catalase activity was determined according to Bergmeyer [49] monitoring hydrogen peroxide decrease at 240 nm. Superoxide dismutase activity was measured as the inhibition of the rate of reduction of cytochrome c by the superoxide radical, observed at 550 nm [50]. Peroxidase activity was measured following the oxidation of pyrogallol at 420 nm [51]. The activity of glutathione peroxidase was determined monitoring NADPH oxidation at 340 nm [52].

### Purification of copper-binding proteins

Supernatants obtained by ultracentrifugation (18,000 *g*, 45 min) of cell extracts were heated at 65°C for 10 min to enrich thermostable proteins, since thermostability is common to several copper-binding proteins [53]. Samples were then centrifugated at 10,000 *g *for 10 min and supernatants purified by affinity chromatography on a Sepharose Chelating resin (Sigma) loaded with 0.2 M CuSO_4 _(copper resin). Samples (ca. 800 μg protein) were incubated for 20 min with 0.5 mL of copper resin previously equilibrated with 1 mL of binding buffer (0.02 M Na_2_HPO_4_, 0.5 M NaCl, pH 7.2). Unbound proteins were removed by gravity flow and the column washed three times with 0.5 mL of binding buffer. Bound proteins were eluted first with 0.02 M Na_2_HPO_4_, 0.5 M NaCl, pH 3.5 and then with 0.02 M Na_2_HPO_4_, 0.5 M NaCl, 0.05 M EDTA, pH 7.2. Proteins from 200 μL of each elution fraction were precipitated with 60 μl of 20% (w/v) TCA, resuspended in 50 μL of SDS-Sample buffer (0.25 M Tris-Cl pH 6.8, 50% (v/v) glycerol, 10% (w/v) sodium dodecyl sulphate, 5% (v/v) β-mercaptoethanol, 0.25% (w/v) bromophenol blue), heated at 99°C for 5 min and applied to 18% (w/v) polyacrylamide gels. Electrophoresis in denaturing conditions (SDS-PAGE) was carried out according to Laemmli [54]. Gels were stained by *GelCode Blue *(Pierce).

### Mass spectrometry

Bands were excised from the polyacrylamide gels, cut into small pieces and de-stained by repeated washing cycles alternating 50 mM ammonium hydrogen carbonate and pure acetonitrile. After complete destaining, gel particles were dehydrated by acetonitrile, covered with trypsin solution (12.5 ng/mL in 50 mM ammonium hydrogen carbonate, pH 8.0) and incubated 1 h on ice. Excess liquid was removed and the gel pieces covered with a solution of 50 mM ammonium hydrogen carbonate (pH 8.0) and incubated overnight at 37°C. Tryptic peptides were extracted by alternating incubation in pure acetonitrile and 1% formic acid. Samples were lyophilised, resuspended in 1% formic acid, and desalted by *ZipTip *(Millipore) before ESI-MS analysis.

ESI-MS experiments were performed with a hybrid Quadrupole-Time-of-Flight (q-TOF) mass spectrometer (*QSTAR ELITE*, Applied Biosystems) equipped with a nano-electrospray ionisation sample source. Metal-coated borosilicate capillaries (Proxeon, Denmark) with medium-length emitter tip of 1-μm internal diameter were used for off-line analysis. The instrument was calibrated with standard solution Renin (MH2+ 879.97 Da and its fragment MH+ 110.07 Da, Applied Biosystems). Peptide identification was performed using the *MASCOT *software with the following parameters: 2 missed cleavages, peptide tolerance 0.6 Da, MS/MS tolerance 0.6 Da, peptide charges 2+ and 3+. Only monoisotopic masses were considered as precursor ions.

Spectra of tryptic peptides were acquired in the 400-1,500 *m/z *range, with 1.0 sec accumulation time, ion-spray voltage 1,3000 V, declustering potential 60 V, with active Information Dependent Acquisition (IDA), using rolling collision energy to fragment peptides for MS/MS analysis.

### Determination of Reactive Oxygen Species

Samples containing 5 × 10^6 ^cells were withdrawn from exponential cultures. Cells were harvested by centrifugation, washed twice with de-ionized water, four times with 10 mM citric acid in 0.5% (w/v) NaCl, once with 1 mL of PBS and finally incubated with 0.5 mL of 5 μM (v/v) dihydroethidium (Sigma, stock solution 30 mM in DMSO) for 30 min in the dark. Stained cells were washed three times with PBS and sonicated for 15 sec. The fluorescence signal was detected using a Cary Eclipse spectrofluorimeter (Varian, CA, USA) at excitation wavelength of 518 nm and emission wavelength of 605 nm. Fluorescence values were normalized against those of not stained cells.

## Competing interests

The authors declare that they have no competing interests.

## Authors' contributions

GMA performed most of the physiological and biochemical characterization of yeasts. SB participated in the design of experiments and in the analysis of results. SP designed and carried out the experiments of cytofluorimetry. BS drew attention on the importance of Cu tolerance in yeast and suggested the experimental strategy of adaptation. ML conceived this study, contributed to the interpretation of results and drafted the paper. All authors read and approved the final manuscript.

## Supplementary Material

Additional file 1 **Table**. Specific growth rate (h^-1^) and final cell density (cells · mL^-1^) ^. ^10^7 ^of non-evolved, evolved and de-adapted cells from *C. humilis *AL5, and *S. cerevisiae *BL7, EL1 and GL6 strains grown on YPD and/or YPD + 2.5 g · L^-1 ^CuSO_4_(Cu). n.d.: not determined. Click here for file

Additional file 2 **Growth of yeast cells in YPD supplemented with 1, 1.5 and 2 g · L^-1 ^CuSO_4_**. Evolved (black circles), non-evolved (black triangles) cells of *S. cerevisiae *BL7 (**a**), *S. cerevisiae *EL1 (**b**), *S. cerevisiae *GL6 (**c**). The values reported are averages of three replicates. For values of standard deviations ≤ 0.6 error bars are not appreciable. Click here for file

Additional file 3 **Intracellular copper measured during growth in YPD + 1 g · L^-1 ^CuSO_4_**. *S. cerevisiae *BL7 (**a**); *S. cerevisiae *EL1 (**b**) and *S. cerevisiae *GL6 (**c**). White bars: evolved cells; grey bars: non-evolved cells. The amount of Cu is reported as mg · g^-1 ^biomass. Values are the mean of three replicates. Click here for file

Additional file 4 **Fluorimetric analysis of superoxide anion (OH^-•^) production in *S. cerevisiae *BL7 growing at different CuSO_4 _concentration**. Detection of OH^-• ^was carried out after growth in YPD and in YPD supplemented with 1, 1.5 and 2 g · L^-1 ^CuSO_4_. White bars: evolved cells; grey bars: non-evolved cells. OH^-• ^formation is expressed as fluorescence intensity of ethidium in arbitrary units. Data presented are the mean of at least three independent analyses. Click here for file
