# Supplementary material for: Laboratory evolution of copper tolerant yeast strains
Source: Microb Cell Fact. 2012 Jan 3;11:1. doi: 10.1186/1475-2859-11-1 (PMC3276424; doi:10.1186/1475-2859-11-1)
Supplement: Additional file 1 — Table. Specific growth rate (h-1) and final cell density (cells · mL-1) . 107 of non-evolved, evolved and de-adapted cells from C. humilis AL5, and S. cerevisiae BL7, EL1 and GL6 strains grown on YPD and/or YPD + 2.5 g · L-1 CuSO4(Cu). n.d.: not determined. [file 1475-2859-11-1-S1.PDF]

	Specific growth rate (h^{-1})						Final cell density [(cells · mL^{-1}) · 10^7]				
	non-evolved		evolved		de-adapted		non-evolved		evolved		de-adapted
	YPD	Cu	YPD	Cu	Cu		YPD	Cu	YPD	Cu	Cu
AL5	0.50	0.10	0.49	0.33	0.40	AL5	20.75	3.60	21.00	25.57	26.23
BL7	0.46	n.d.	0.45	0.24	0.31	BL7	24.03	n.d.	24.11	21.97	16.10
EL1	0.50	n.d.	0.48	0.38	0.40	EL1	13.37	n.d.	13.40	10.98	9.95
GL6	0.54	n.d.	0.53	0.39	0.43	GL6	10.40	n.d.	10.45	8.40	7.84
