# Supplementary figures and images for: Laboratory evolution of copper tolerant yeast strains
Source: Microb Cell Fact. 2012 Jan 3;11:1. doi: 10.1186/1475-2859-11-1 (PMC3276424; doi:10.1186/1475-2859-11-1)

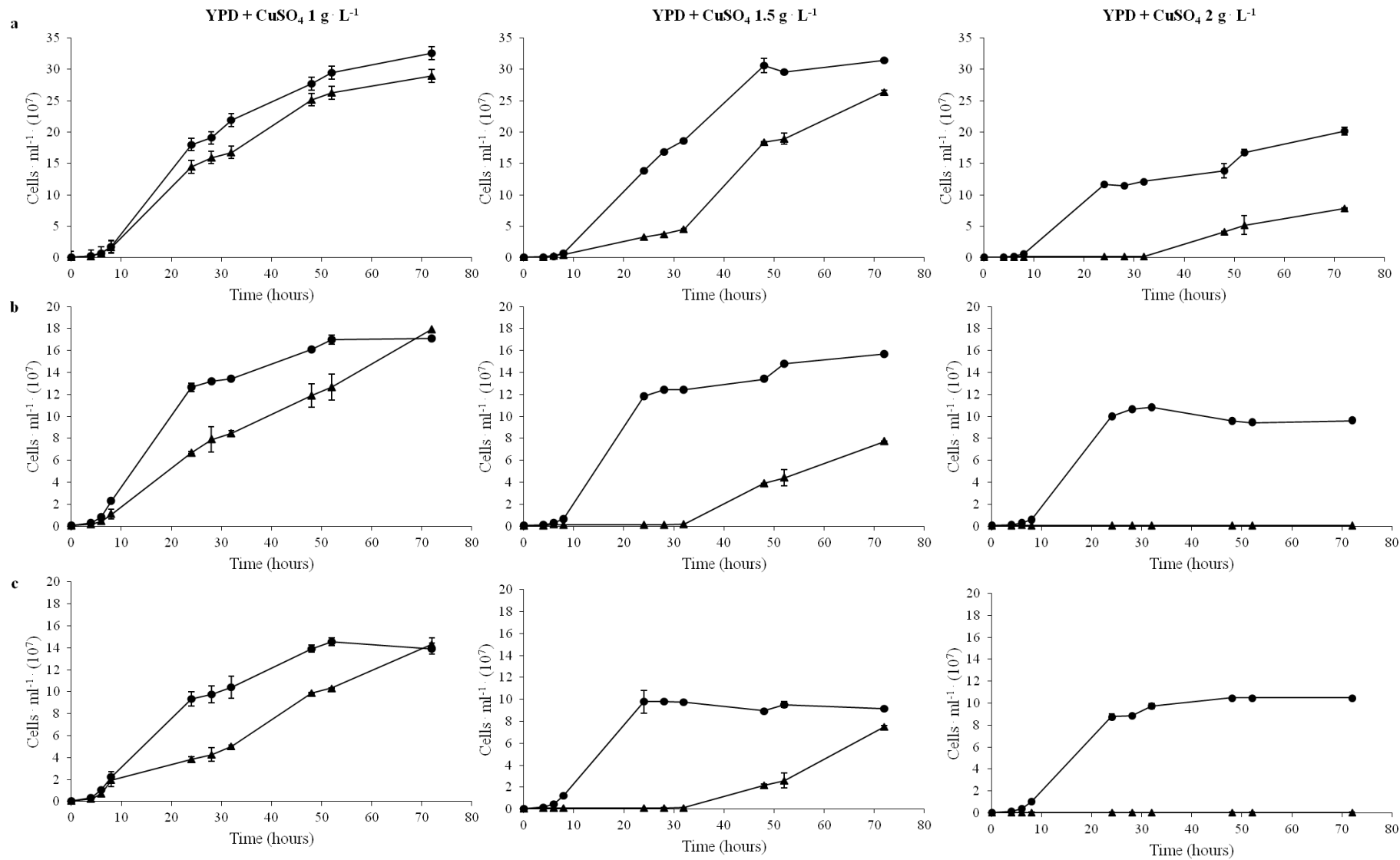

Supplement: Additional file 2 — Growth of yeast cells in YPD supplemented with 1, 1.5 and 2 g · L-1 CuSO4. Evolved (black circles), non-evolved (black triangles) cells of S. cerevisiae BL7 (a), S. cerevisiae EL1 (b), S. cerevisiae GL6 (c). The values reported are averages of three replicates. For values of standard deviations ≤ 0.6 error bars are not appreciable. [file 1475-2859-11-1-S2.PDF]

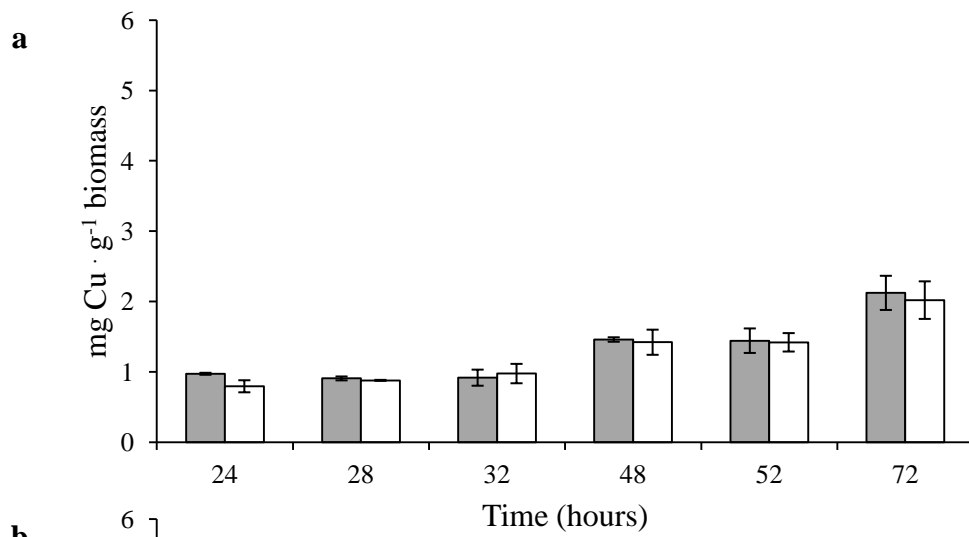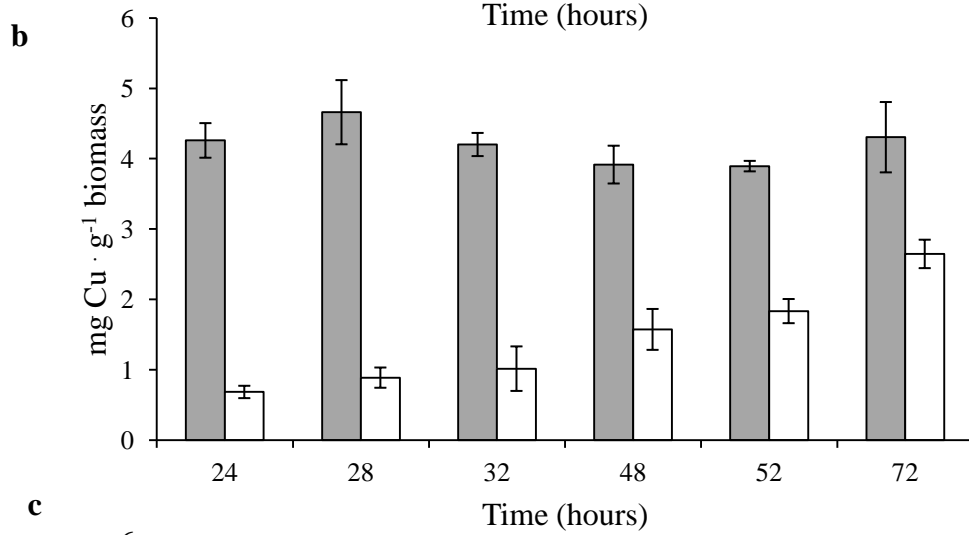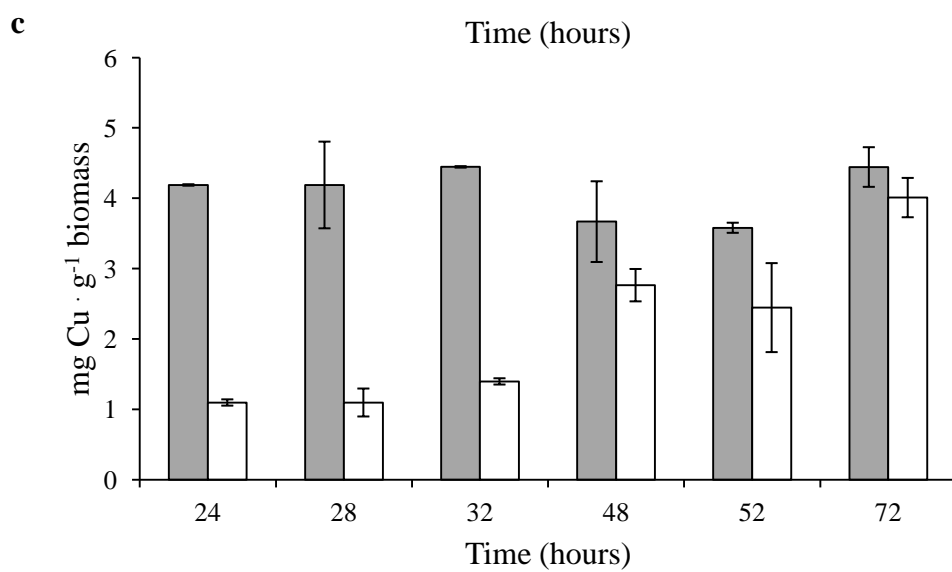

Supplement: Additional file 3 — Intracellular copper measured during growth in YPD + 1 g · L-1 CuSO4. S. cerevisiae BL7 (a); S. cerevisiae EL1 (b) and S. cerevisiae GL6 (c). White bars: evolved cells; grey bars: non-evolved cells. The amount of Cu is reported as mg · g-1 biomass. Values are the mean of three replicates. [file 1475-2859-11-1-S3.PDF]

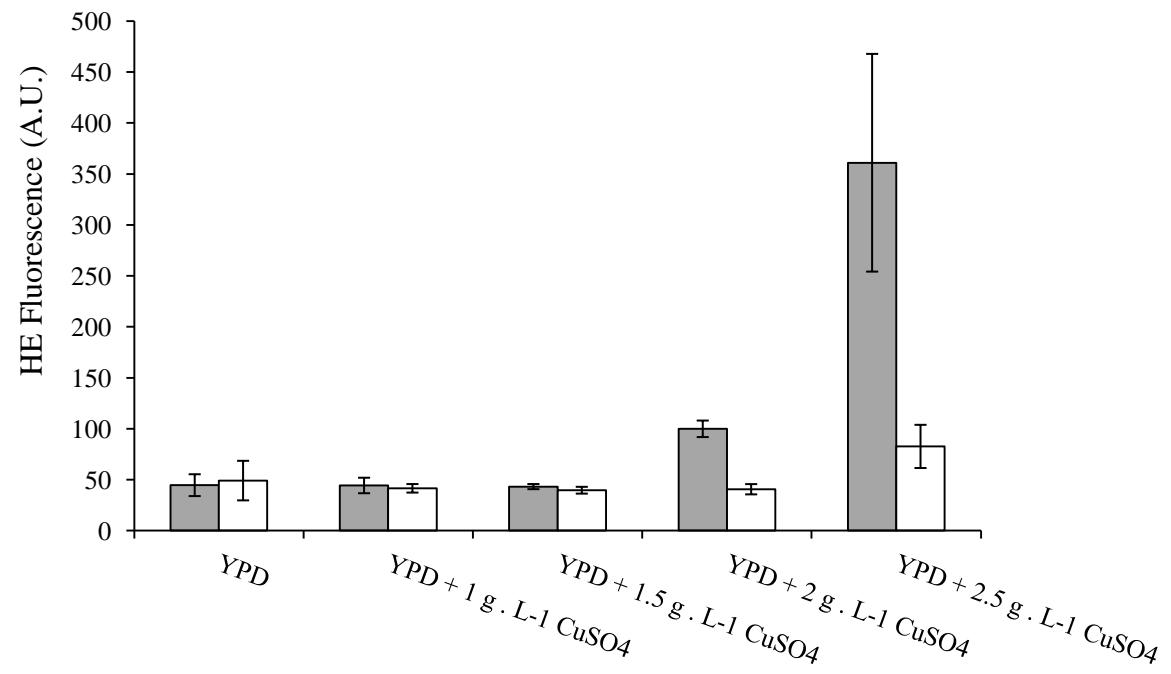

Supplement: Additional file 4 — Fluorimetric analysis of superoxide anion (OH-•) production in S. cerevisiae BL7 growing at different CuSO4 concentration. Detection of OH-• was carried out after growth in YPD and in YPD supplemented with 1, 1.5 and 2 g · L-1 CuSO4. White bars: evolved cells; grey bars: non-evolved cells. OH-• formation is expressed as fluorescence intensity of ethidium in arbitrary units. Data presented are the mean of at least three independent analyses. [file 1475-2859-11-1-S4.PDF]
